# Supplementary material for: Comprehensive Metabolomic Comparison of Five Cereal Vinegars Using Non-Targeted and Chemical Isotope Labeling LC-MS Analysis
Source: Metabolites. 2022 May 10;12(5):427. doi: 10.3390/metabo12050427 (PMC9144210; doi:10.3390/metabo12050427)

Supplemental Figure S2. Some details of main metabolites in the context were annotated by SIRIUS4, GNPs and MS-FINDER

Metabolite name: N-(1-Deoxy-1-fructosyl)phenylalanine; MS-DIAL ID:pos:12437; neg: 25824

Adduct type:[M+H]$^+$ (MS DIAL ID:12437)

SIRIUS4 result:

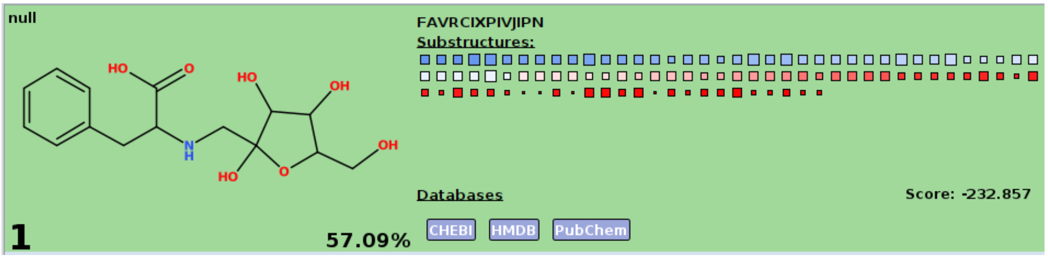

GNPs result (Cosine:0.75):

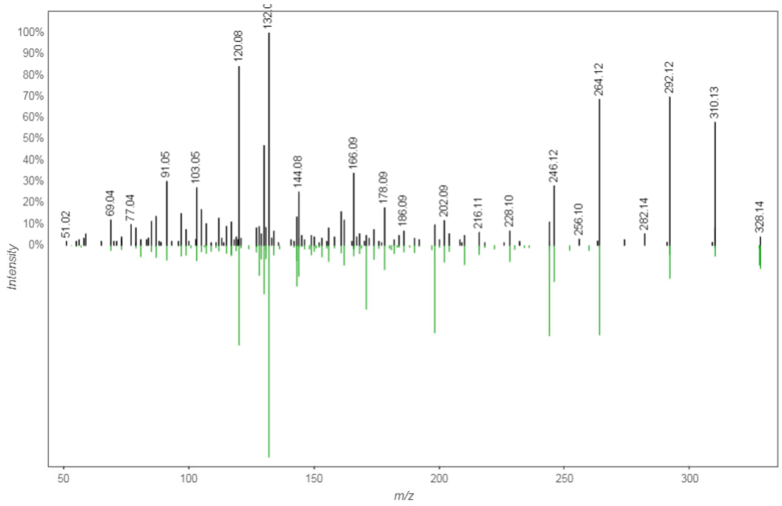

MS-FINDER result:

Adduct type:[2M-H]$^-$ (MS DIAL ID: 25824)

N-(1-Deoxy-1-fructosyl)phenylalanine was the top 1 candidate metabolite predicted by MS-FINDER in negative mode.

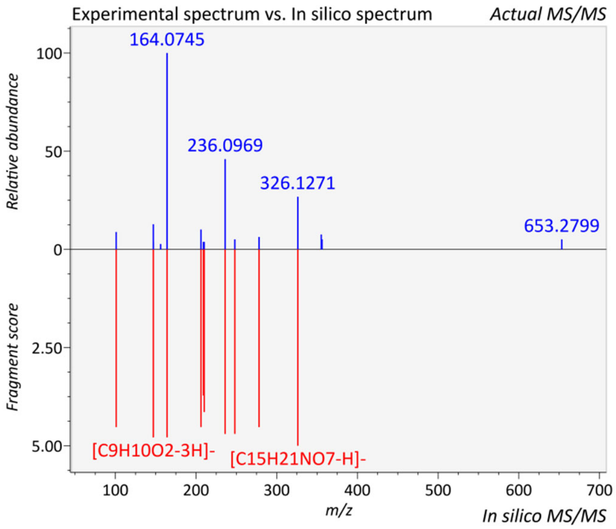

Metabolite name: Cyclo(Pro-Leu); MS-DIAL ID: pos:4088

Adduct type:[M+H]$^{+}$ (MS DIAL ID:4088)

SIRIUS4 result:

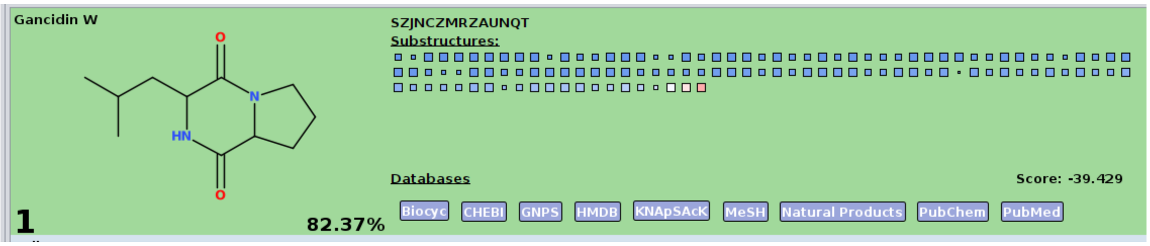

GNPs (Gold) (Cosine:0.87):

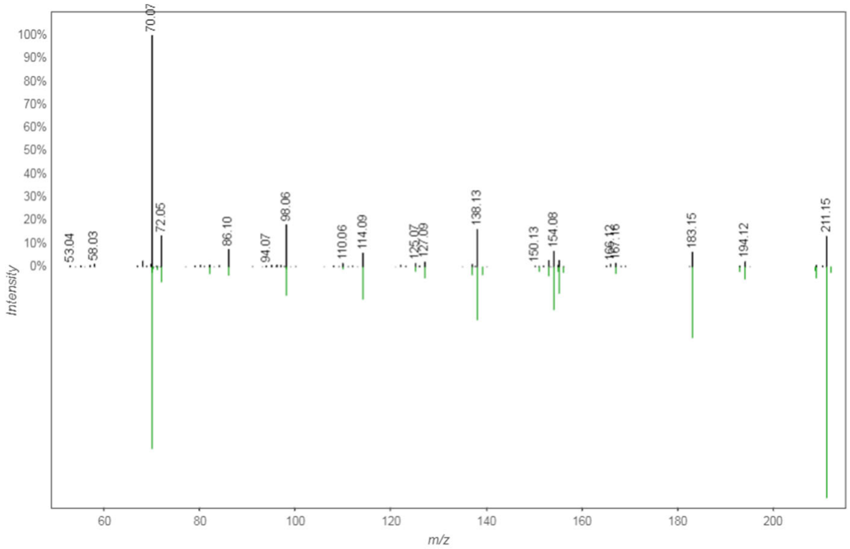

Metabolite name: Cyclo(Phe-Pro); MS-DIAL ID: pos:6200

Adduct type:$[M+H]^+$ (MS DIAL ID:6200)

SIRIUS4 result:

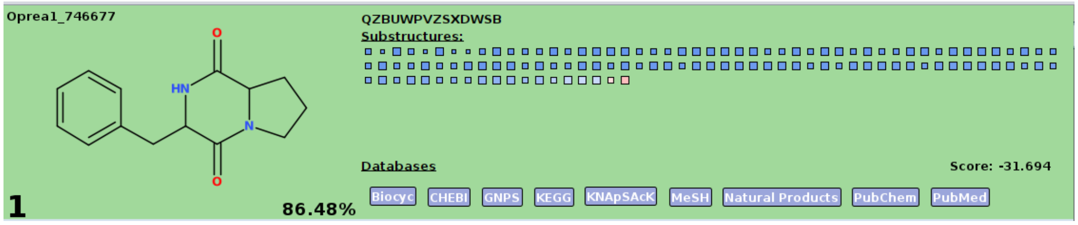

GNPs(Bronze) (Cosine: 0.98):

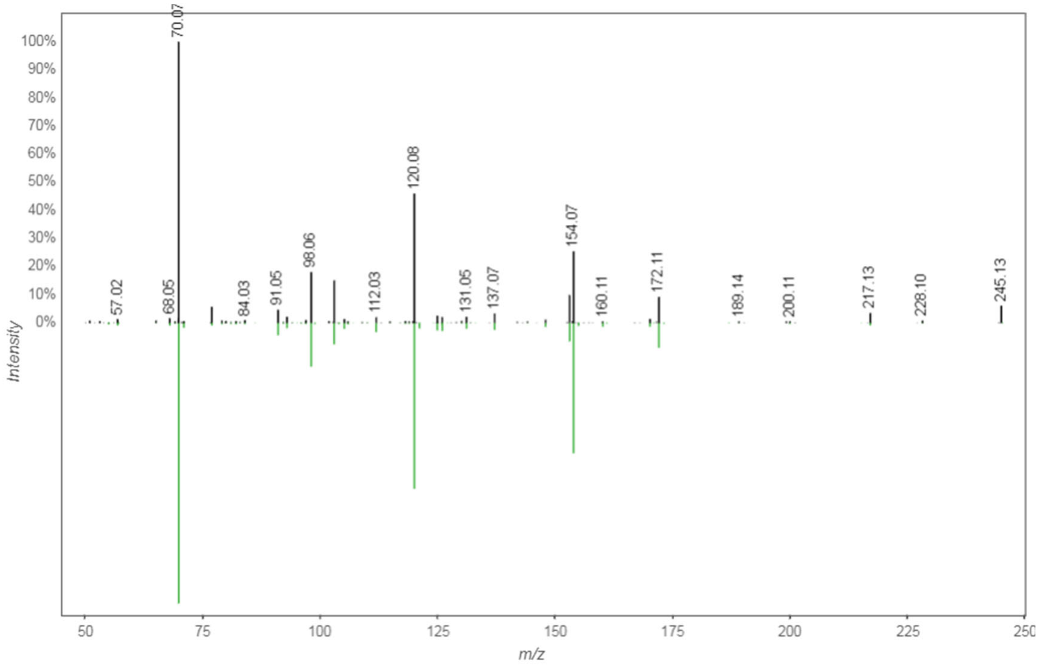

Metabolite name: 9,10,13-Trihydroxystearic acid; MS-DIAL ID:pos:12937; neg:8497

Adduct type:$[M+H]^+$ (MS DIAL ID:12937)

SIRIUS4 result:

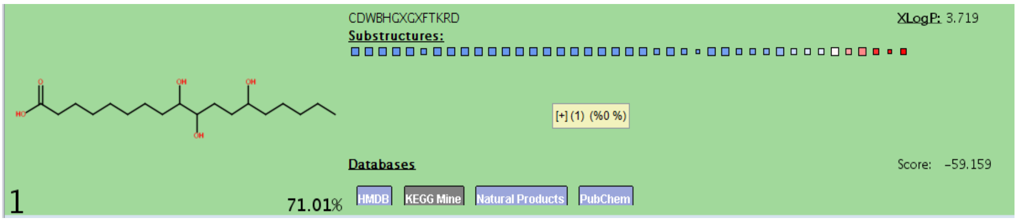

Adduct type: $[M-H]^-$ (MS DIAL ID:8497)

SIRIUS4 result:

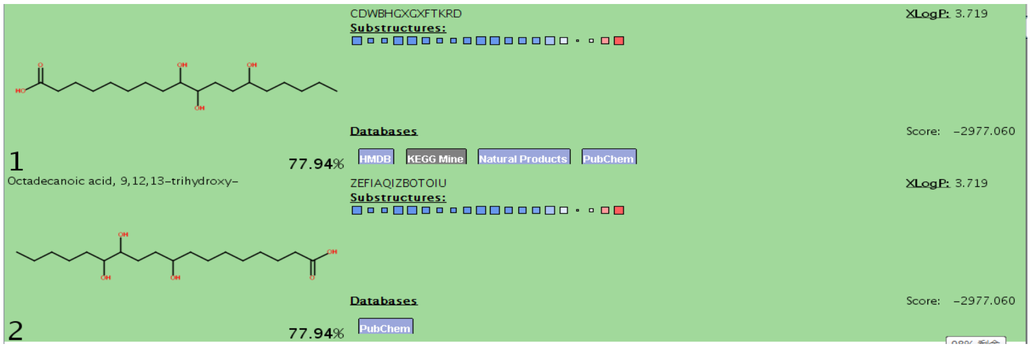

Metabolite name: Tetrahydroharman-3-carboxylic acid; MS-DIAL ID:pos:5332, neg:3433

Adduct type:[M+H]$^{+}$

SIRIUS4 result:

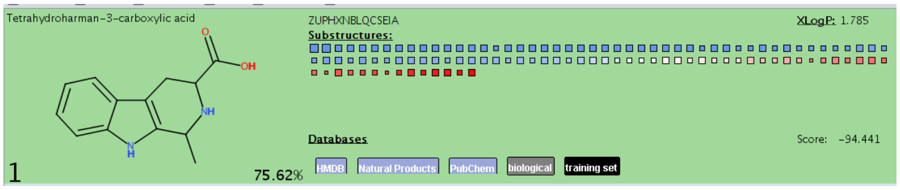

MS-FINDER result:

Tetrahydroharman-3-carboxylic acid was the top 1 candidate metabolite predicted by MS-FINDER in positive mode.

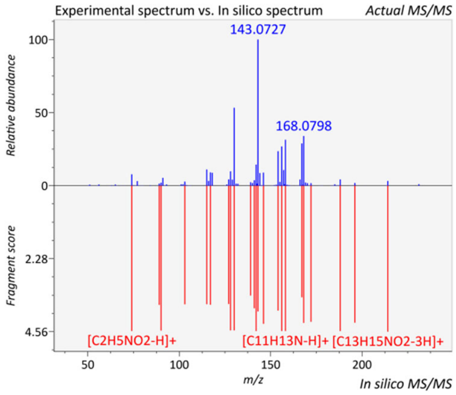

Adduct type: [M-H]$^{-}$

SIRIUS4 result:

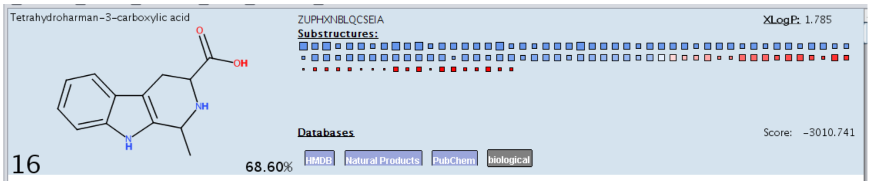

MS-FINDER result:

Tetrahydroharman-3-carboxylic acid was the top 1 candidate metabolite predicted by MS-FINDER in negative mode.

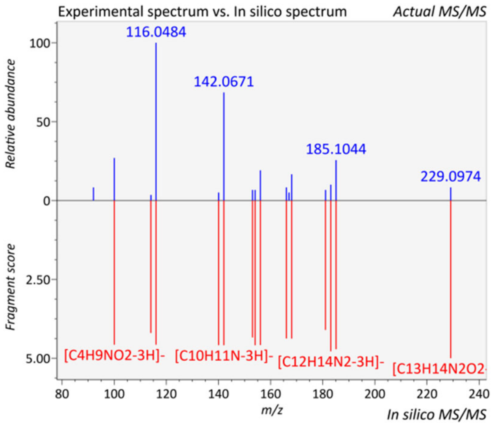

Metabolite name: Harmalan; MS-DIAL ID:pos:2621

Adduct type: [M+H]$^{+}$

SIRIUS4 result:

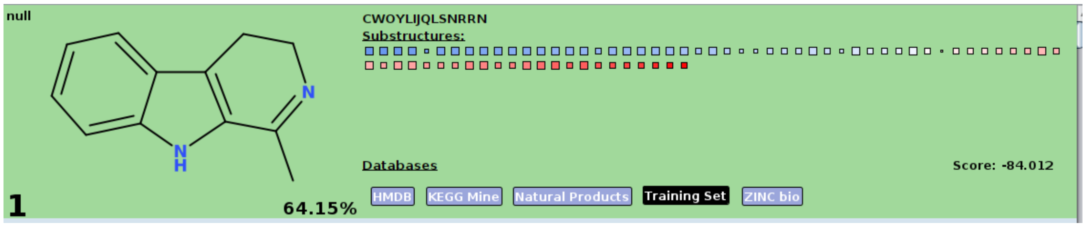

GNPs (Gold) (Cosine: 0.83):

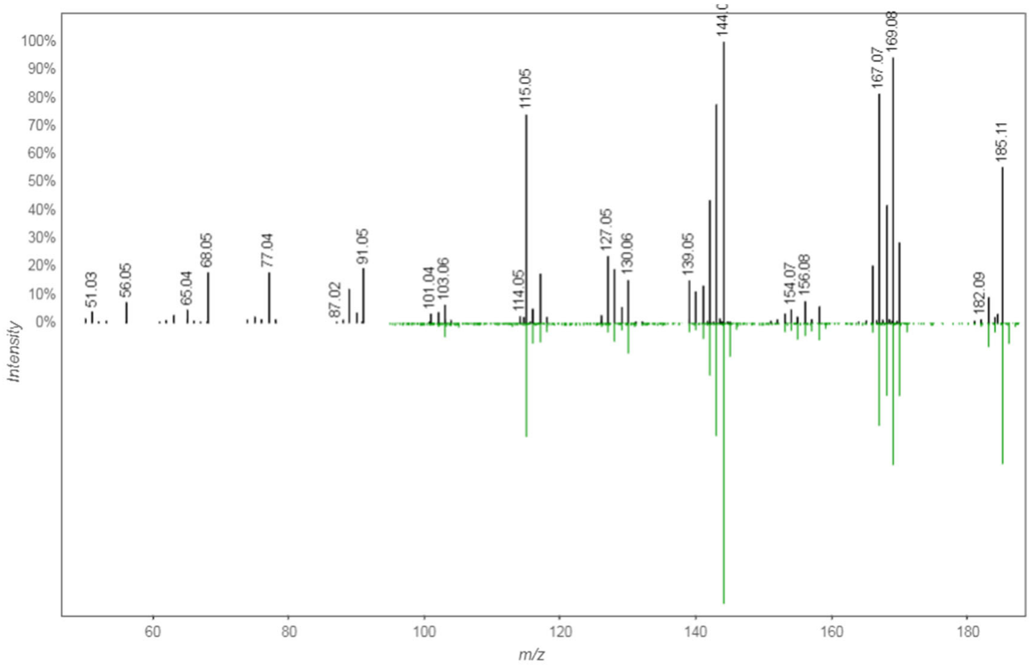

Metabolite name: 3-Phenyllactic acid; MS-DIAL ID: neg:1319,8448

Adduct type: [M-H]⁻

SIRIUS4 result:

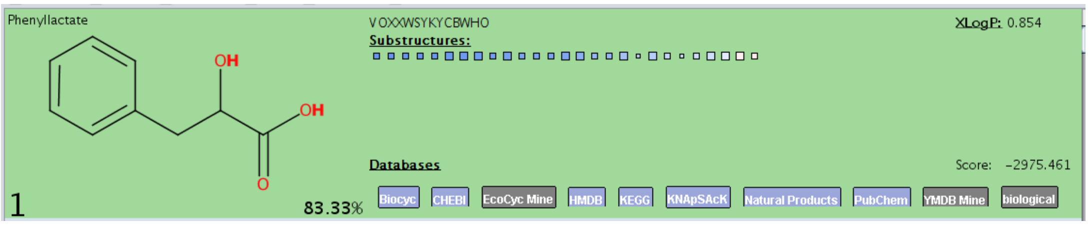

Adduct type:[M-H]⁻ (MS-DIAL ID: neg:1319**).** 3-Phenyllactic acid was the top 1 candidate metabolite predicted by MS-FINDER in negative mode.

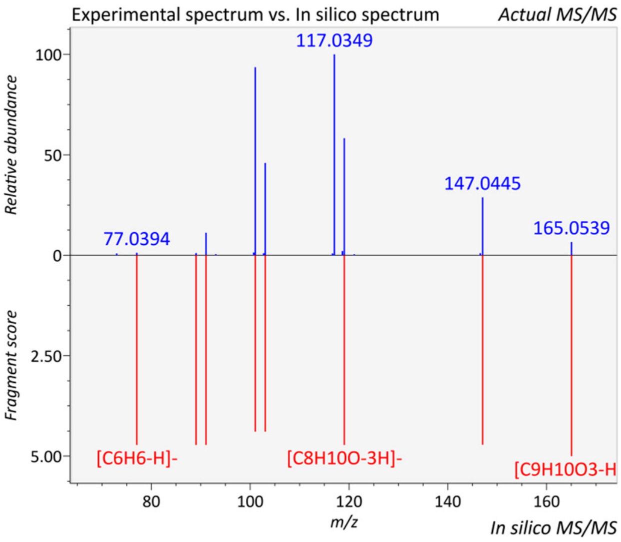

Adduct type:[2M-H]⁻ (MS-DIAL ID: neg:8448**).** 3-Phenyllactic acid was the top 1 candidate metabolite predicted by MS-FINDER in negative mode.

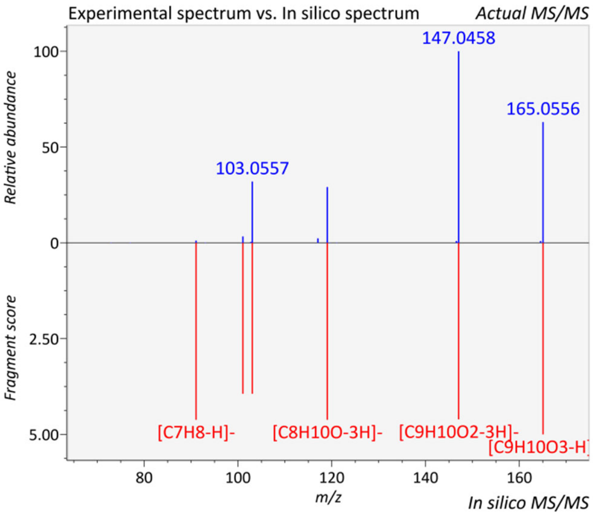

Metabolite name: Hydroxyphenyllactic acid; MS-DIAL ID:neg:1702
SIRIUS4 result:

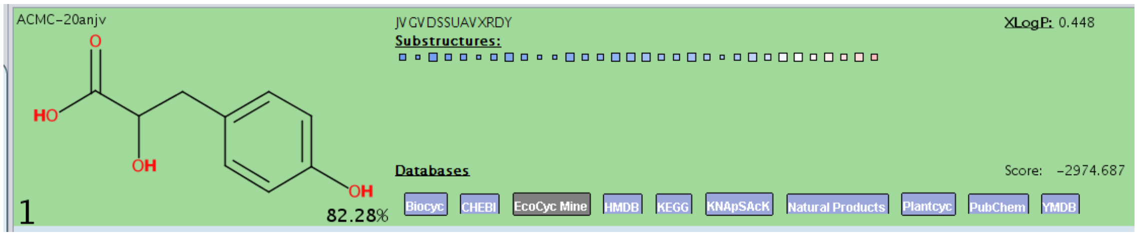

Adduct type:[M-H]$^-$ (MS-DIAL ID: neg:1702**).** Hydroxyphenyllactic acid was the top 2 candidate metabolite predicted by MS-FINDER in negative mode.

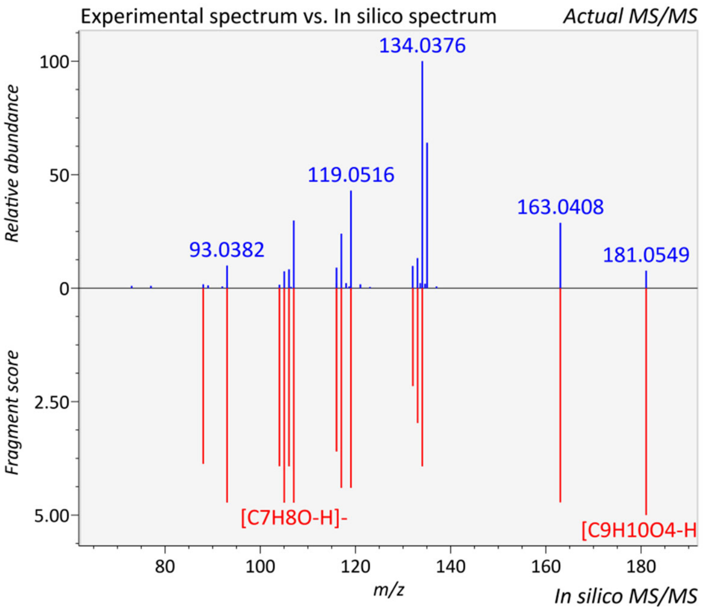

Supplement: Supplementary file 1 [file metabolites-12-00427-s001.zip › Supplemental Figure S2.pdf]
