# Supplementary figures and images for: Comprehensive Metabolomic Comparison of Five Cereal Vinegars Using Non-Targeted and Chemical Isotope Labeling LC-MS Analysis
Source: Metabolites. 2022 May 10;12(5):427. doi: 10.3390/metabo12050427 (PMC9144210; doi:10.3390/metabo12050427)

## Slide 1
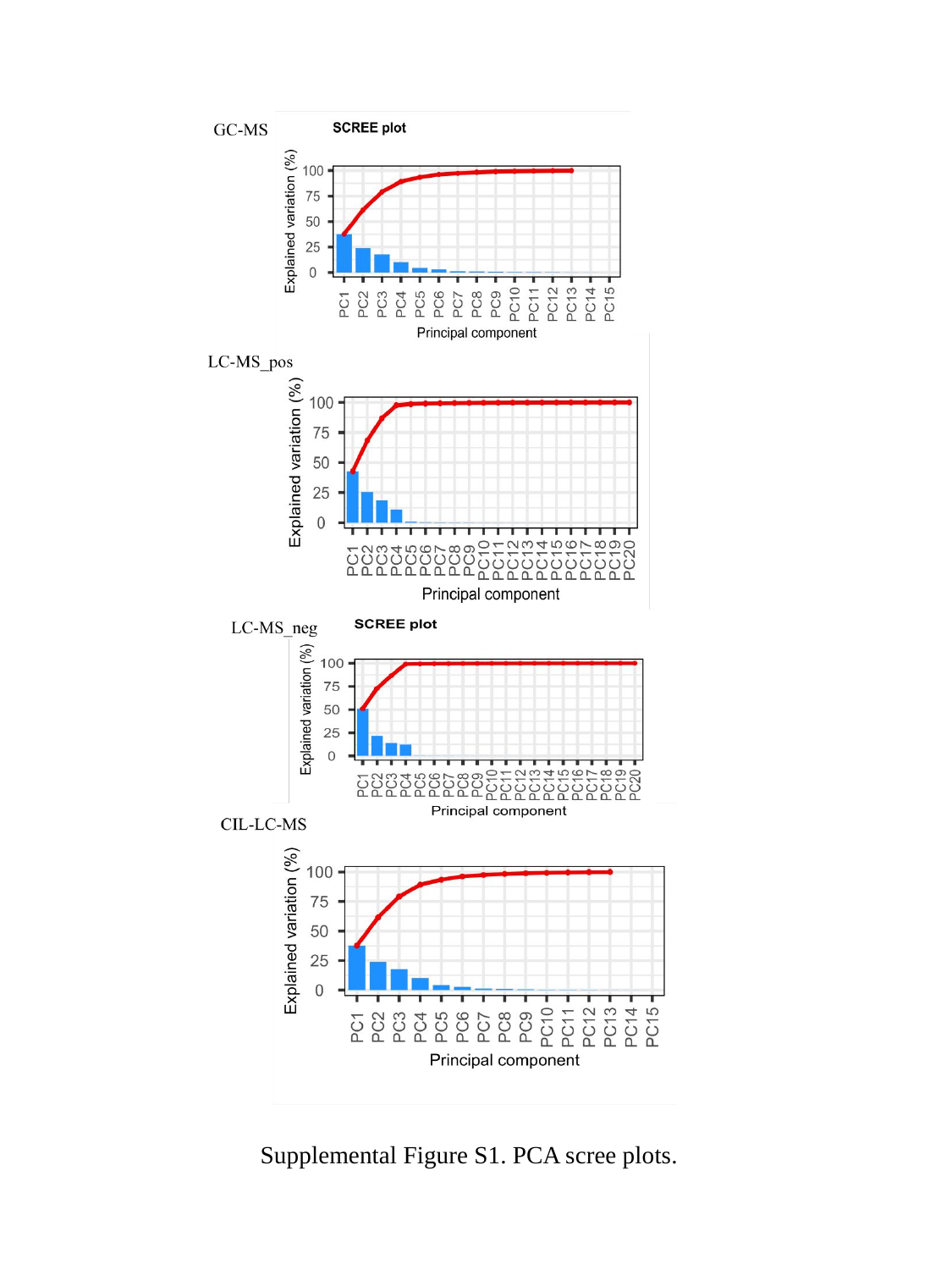

Supplemental Figure S1. PCA scree plots.

Supplement: Supplementary file 1 [file metabolites-12-00427-s001.zip › Supplemental Figure S1.pptx]
